# Rule-based multi-scale simulation for drug effect pathway analysis

**DOI:** 10.1186/1472-6947-13-S1-S4

**Published:** 2013-04-05

**Authors:** Woochang Hwang, Yongdeuk Hwang, Sunjae Lee, Doheon Lee

**Affiliations:** 1 Department of Bio and Brain Engineering, KAIST, Daejeon, South Korea

## Abstract

**Background:**

Biological systems are robust and complex to maintain stable phenotypes under various conditions. In these systems, drugs reported the limited efficacy and unexpected side-effects. To remedy this situation, many pharmaceutical laboratories have begun to research combination drugs and some of them have shown successful clinical results. Complementary action of multiple compounds could increase efficacy as well as reduce side-effects through pharmacological interactions. However, experimental approach requires vast cost of preclinical experiments and tests as the number of possible combinations of compound dosages increases exponentially. Computer model-based experiments have been emerging as one of the most promising solutions to cope with such complexity. Though there have been many efforts to model specific molecular pathways using qualitative and quantitative formalisms, they suffer from unexpected results caused by distant interactions beyond their localized models.

**Results:**

In this work, we propose a rule-based multi-scale modelling platform. We have tested this platform with Type 2 diabetes (T2D) model, which involves the malfunction of numerous organs such as pancreas, circulation system, liver, and adipocyte. We have extracted T2D-related 190 rules by manual curation from literature, pathway databases and converting from different types of existing models. We have simulated twenty-two T2D drugs. The results of our simulation show drug effect pathways of T2D drugs and whether combination drugs have efficacy or not and how combination drugs work on the multi-scale model.

**Conclusions:**

We believe that our simulation would help to understand drug mechanism for the drug development and provide a new way to effectively apply existing drugs for new target. It also would give insight for identifying effective combination drugs.

## Background

Over past decades, the drug discovery process has been slowed down and the costs for developing a drug have gone up [1]. It is because experimental drug discovery has focused on phenotype result without underlying mechanism. The underlying mechanisms of many of drugs are still unascertainable like a black box [2]. Therefore, it is difficult to identify off-targets of drugs, which cause unexpected side-effects.

Recently, the development of biology technological advancements increased the understanding of molecular biology. It makes possible to extend our knowledge of mechanisms of drugs in a molecular level.

Accumulated large number of observed data of a molecular behaviour makes possible to construct computational drug-response prediction model. The computational model brought benefits such as time reduction, cost reduction and side-effects prediction to drug development.

The drug response accompanies the change in effect on cellular level to organ level caused by a drug. Therefore, computational model for drug response prediction is needed to be represented with multi-level interactions [3].

Systems approaches have long been used in pharmacology to understand drug action at the organ and organismal levels using experimental and computational approaches It would be great challenges to construct a computational model of a multi-level for understanding drug action and discovering drug with the lack of multi-level data.

Drug response prediction model can be used to predict the efficacy of multi-compound drug as well as the efficacy of single-compound drug [4-6]. Complex disease such as cardiovascular disease, diabetes, and cancer, are caused by complex factors. To treat complex disease, multi-compound drug is more efficacious than single-compound drug. For example, in a case of recently FDA-approved CLEOPATRA that targets *HER*2-positive breast cancer, the addition of pertuxumab significantly increased progression-free survival time, compared with trastuzumab plus docetaxel. Also it has no increment in cardiac toxic effects, which is the side-effect of trastuzumab plus docetaxel [7].

The development of multi-compound drugs using cell-based experiments is quite challenging because of vast amounts of possible combinations in pharmaceutical laboratories. For instance, about 100,000 potential therapeutic agents have been the object of focus to be tested in NCI60 anticancer drug screen [8]. If pair of combination drugs are tested among them, the number of screening is more than. When we screen combinations of more than two drugs or multi-dose, the number of screening is increasing exponentially [9]. Computational approaches have emerged as one of the most promising solutions to this challenge. To simulate the behaviour of dynamic system caused by multi-compound drugs, it is needed to systematically model a human body as multi-level model.

### Computational model for drug response simulation

The simulation model of biological networks could be differed according to the specification of nodes in a system. Nodes that are specified by discrete values are simulated by qualitative method such as Boolean network or Petri-net model. Others nodes that have continuous values are simulated by quantitative method such as ordinary differential equation (ODE) model.

Jack et al. conducted qualitative investigation of cellular responses using Boolean network [10]. They investigated the response of biological systems by the effect of chemicals at molecular level and tissue level. Jin et al. developed enhanced Petri-net model to investigate a genetic mechanism for effects of drug combinations [11]. They integrated data on large-scale biological network and made Petri-net model from the biological network. Although their model is large enough to investigate drug actions, it predicts only cellular response of pairwise drug combinations by Petri-net simulation result.

To obtain accurate results in analysing drug action by biological networks, constructing an accurate biological network is needed. Nelander et al. made a model that derives network models from molecular profiles of perturbed cellular systems [12]. They predicted results of multiple genetic alterations by ODE model.

Previous research focused on constructing models that investigate the biological response of the drug effect. However, previous research did not investigate at whole-body level.

### Multi-scale modelling

Type 2 diabetes (T2D) is caused by a problem in the way body makes or uses insulin [13]. Usually when we get T2D, our fat, liver, and muscle cells do not respond properly to insulin. T2D cannot be understood by investigating only one organ, which produces insulin for drug discovery processing. It occurs when several organs' interaction does not work properly. So, we need to investigate systematically organs' interactions. There are many complex diseases that have multi causalities, such as schizophrenia, stroke, etc.

**Figure 1:**
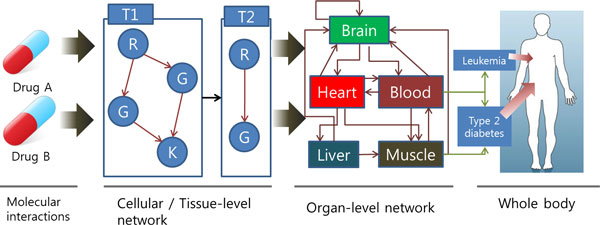
**The scheme of the multi-scale network**. Molecular interaction between drug and target lead to alterations in cellular- and tissue-level network, which lead to alteration in organ-level network, which, in turn, lead to whole body phenotype change.

To investigate these complex diseases, we need to model biological systems as multi-level system that contains molecular level, cell/tissue-level and organ-level. Cell-level are affect by biological processes at the molecular level and biological processes at the cell/tissue level influence dynamics at organ level as Figure 1. This hierarchical organization and the causalities between different levels are characteristics of biological systems [14,15]. As we mentioned, we are lack of understanding about tissue level and organ level. Hence, it is difficult to make a whole-body model mathematically, like in ODE. Qualitative modelling could be used to overcome this difficulty. Among qualitative modelling methods, we will utilize the context of rule-based modelling to model a whole-body level.

Simpler form of rule-based modelling [16,17] makes it possible to converts rules based on various sources of knowledge, such as literature and computational models of different formalisms. And this formalism can describe more diverse states of a component in biological system than the Boolean network model.

We previously proposed rule-based multi-level simulation model [18]. In previous work, we showed the possibility of our model to simulate the multi-compound drug effect on human body, which is multi-level system. In this work, we improved previous model to have better performance.

## Results

We have collected 190 rules from various sources such as pathway database, ODE model, and Petri-net model. The number of rules extracted from pathway databases, ODE models, and Petri-net models are 67, 5 and 45 respectively.

In this work, we have focused on type 2 diabetes (T2D) as a target for the simulation. Therefore, we have curated T2D-related rules from a pathway database, such as Kyoto Encyclopedia of Genes and Genomes (KEGG), Pathway Interaction Database (PID) and related literatures. These rules have been extracted from pathway data that contain known twenty-two T2D drugs: Saxagliptin, Sitagliptin, Vildagliptin, Metformin, Miglitol, Voglibose, Acarbose, Exenatide, Liraglutide, Mitiglinide, Nateglinide, Repaglinide, Chlorpropamide, Glipizide, Gliquidone, Tolbutamide, Glimepiride, Glyburide, Pioglitazone, Rosiglitazone, Troglitazone, Pramlintide [19].

In this work, We have also integrates converted rules from different formalism like T2D-related nonlinear ODE model. This ODE model contains beta-cell mass, insulin, glucose as variables. Components, which are the features describing pertained organs, in converted rules are beta-cell, hepatocyte, adipocyte and circulation system. That is, these are the T2D-related organs or cell types.

The initial conditions of T2D related attributes are known from literature [13]. We set the initial conditions of others as normal. Initial states of all attributes in our model are normal. To simulate effect of T2D drugs with our model, we need to make our model to T2D model. To make our model as T2D model, we have added T2D-cause rules, which are related to insulin resistant [20-22]. During simulation, rules induced by T2D drugs and rules caused by T2D compete each other.

We simulated effect of well-known twenty-two T2D drugs. The criterion of T2D diagnosis is established by the World Health Organization definition, which is the state of glucose in blood.

### Drug effect pathway

**Figure 2:**
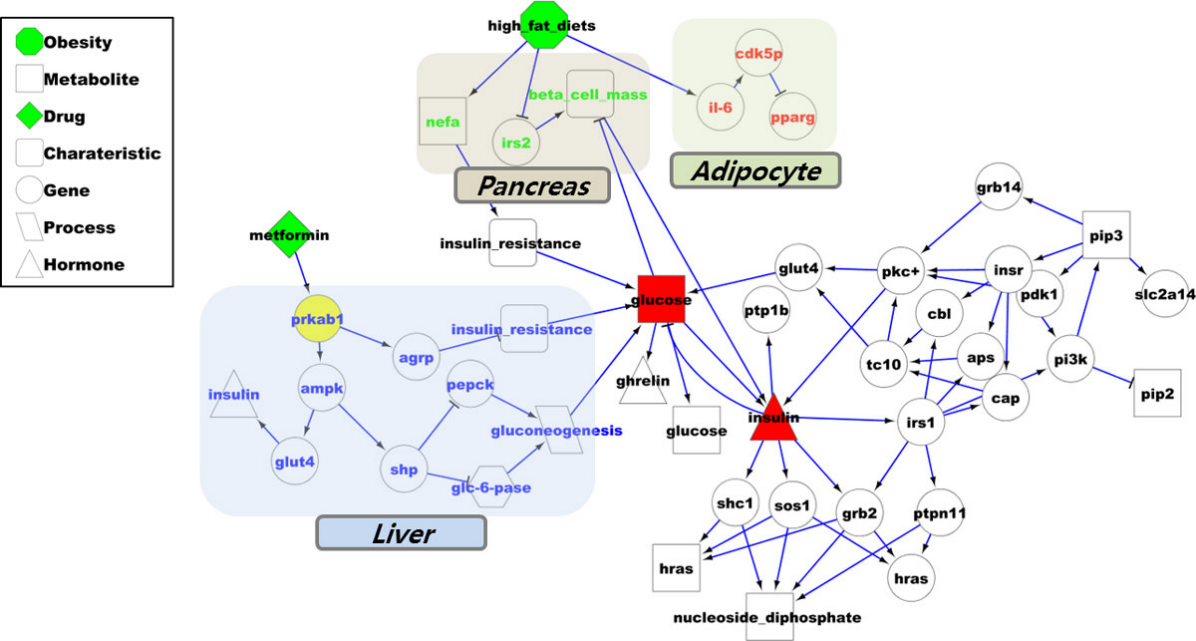
**Single drug simulation result on T2D model**. Metformin drug effect pathway on T2D model.

Our simulation platform shows drug effect pathways, where the drug actions propagates from the target to the final nodes for the therapy that can change disease state to normal state. Figure 2 demonstrates the metformin effect pathway on T2D model. It shows integrated drug effect pathway of metformin in the rules [20].We simulated effect of single drug at each simulation step among twenty-two drugs. According to simulation results, all twenty-two drugs repress the state of glucose in the circulation system. It could also investigate effects of multi-drugs through a simulation.

According to the metformin effect pathway (Figure 2), metformin targeted PRKAB1 enzyme in hepatocyte. The metformin activate insulin secretion through GLUT4 and inhibits gluconeogenesis, which generate glucose, by inhibiting PEPCK and Glucose 6-phosphatase. This result shows corresponding mechanisms of metformin, which previously studied [23]. Drugs (i.e. pioglitazone, rosiglitazone, troglitazone) targeting muscle cells inhibit insulin resistance and glucose level. Drugs targeting pancreas cells (i.e. exenatide, glyburide etc.) help insulin secretion and regulation of glucose metabolism.

### Combination drug

Our simulation platform can show whether combination drugs have substantial efficacy for treatment or not, and how combination drugs work on multi-scale model. We simulated metformin, which targets hepatocyte, with other drugs as combination drug. There were twenty-one pairs of combination drug that consisted with metformin. It was identified that the sixteen among twenty-one pairs of combination drug have efficacy through our simulation. Fifteen pairs of combination drug have strong evidence of efficacy from previous clinical studies (Table 1).

**Table 1:** Supporting evidence for combination drug simulation result.

Drug A	Drug B	Drug development State
Metformin	Saxagliptin	FDA approved
Metformin	Sitagliptin	FDA approved
Metformin	Vildagliptin	FDA approved
Metformin	Miglitol	Literature [26]
Metformin	Voglibose	FDA approved
Metformin	Liraglutide	Phase 3
Metformin	Mitiglinide	Phase 3
Metformin	Nateglinide	Literature [27]
Metformin	Repaglinide	FDA approved
Metformin	Glipizide	FDA approved
Metformin	Gliquidone	Literature [28]
Metformin	Glimepiride	FDA approved
Metformin	Glyburide	FDA approved
Metformin	Pioglitazone	FDA approved
Metformin	Rosiglitazone	FDA approved

We simulated twenty-one pairs of combination drug that consisted with metformin. It was identified that the sixteen among twenty-one pairs of combination drug have efficacy. Fifteen pairs of combination drug have strong evidence of efficacy from previous clinical studies: ten pairs are FDA-approved and two pairs are in clinical trials. And also three pairs have been investigated in several literatures [26-28].

**Figure 3:**
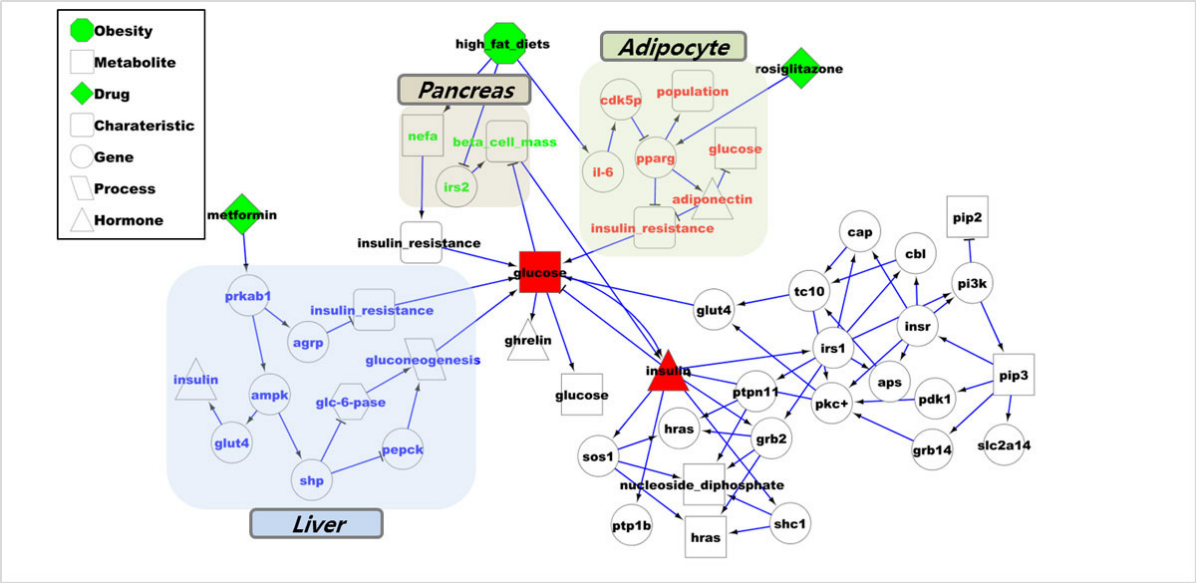
**combination drug simulation result on T2D model**. Combination drug (Metformin and rosiglitazone) effect pathway on T2D model.

In a case of metformin and rosiglitazone (Figure 3), metformin makes the state of gene PRKAB1 in hepatocyte cell up and rosiglitazone makes the state of PPARγ in adipocyte up. The result of metformin action reduces glucose level in circulation and the result of rosiglitazone action reduces glucose level by different pathways. In this way, we can assist to investigate the mechanism of combination drugs by our simulation result.

## Conclusions

In this paper, we proposed novel rule-based whole-body level simulation platform. We defined composition of rules and components of this rule-based platform, which can be regarded as nodes in a network, that is, a multi-level network. Subsequently, we extracted rules by manual curation from literatures, pathway databases and converting different modelling formalisms such as Petri-net and ODE. We modelled multi-scale system that each component and rule has a different time scale of the response. To reflect this biological character, we utilized the threshold to limit the change of component's state for discerning different time-scale. To simulate drug effect on the model, we added curated rules known as causes of T2D. Consequently, there exists an intervention between action of disease-causing rules and action of drug-induced rules. The results of our simulation show drug effect pathways of T2D drugs and how combination drugs work on whole-body level.

The result of simulation shows conceptual effect pathways. In combination drug effect simulation, it can show up whether combination drugs effect on the disease or not. The limitation of prediction of type of combination drugs, such as the synergistic and the addictive would be remained to challenges in this field.

We expect that we can suggest new pairs of combination drugs or mechanisms of drugs by the result of our simulation by augmented rules through text mining approach [24] and other strategies.

## Methods

A whole-body scale platform, which enables us to identify the spread of drug effect to cells or organs, is necessary for whole-body simulation. We have implemented the platform based on rule-based modelling.

### Components and rules

**Figure 4:**
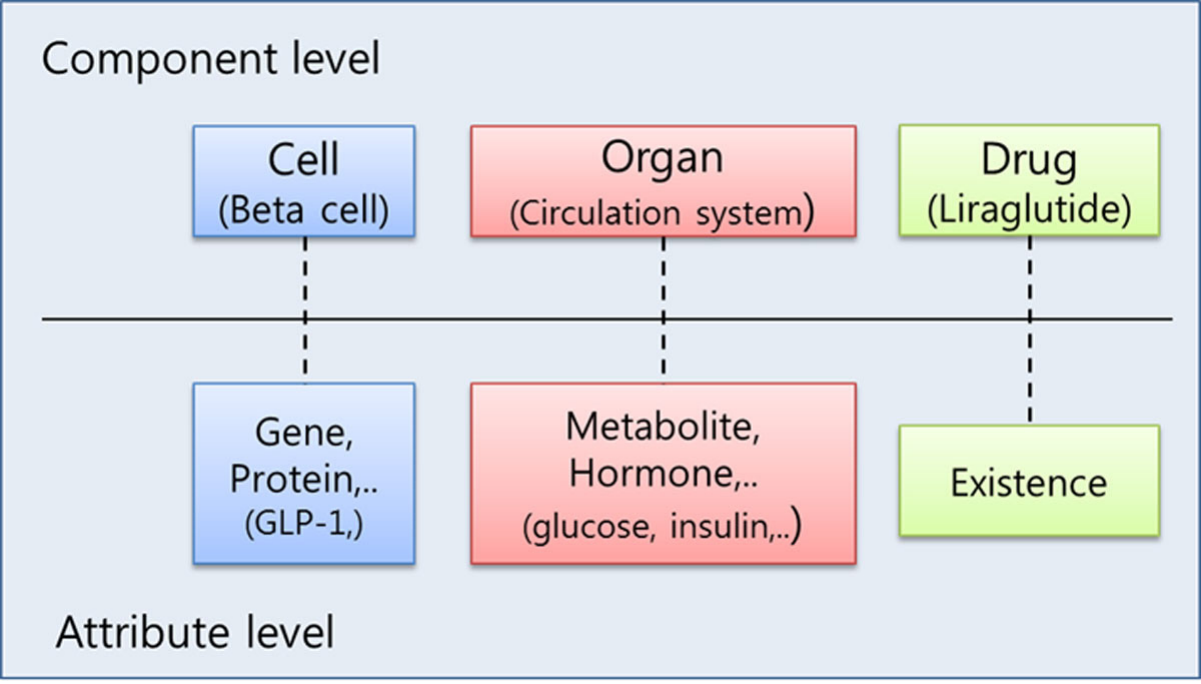
**Component and attribute**. Component is the place where attribute is or drug is. Attribute is the subject of the rule or drug existence.

The description of a biological system in this work consists of a collection of components and rules. A component in a biological system represents an organ, a cell, or a drug and so on (Figure 4). A rule is composed of left and right side to describe the action of rule:

$$\left(\text{Component}\;\text{type},\;\text{Component}\;\text{name},\;\text{Attribute}\;\text{type},\;\text{Attribute}\;\text{name},\;\text{Condition}\right)\to \left(\text{Component}\;\text{type},\;\text{Component}\;\text{name},\;\text{Attribute}\;\text{type},\;\text{Attribute}\;\text{name},\;\text{Action}\right)$$

The left-hand-side of rules is a trigger, which determines the change of the right-hand-side of rules, and the right-hand-side of rules is an expected perturbed status by the left-hand-side. Each side of rule has five features: four features describe the component itself and one feature describes the dynamic status. Component type stands for the kind of components such as organs, cells, or drugs. It makes us to discriminate the hierarchy of components. Component name indicates the specification of corresponding component type. A component can have multiple attributes. Attributes have hierarchical relationship to the component (Figure 4). For example, when component type is a cell, attribute type can be things in a cell such genes, hormones, metabolites and so on. Each attribute has different state values. For example:

$$\begin{array}{c} \text{The}\;\text{states}\;\text{of}\;\text{gene},\;\text{metabolite}=\left\{low,\;normal,\;high\right\}\\ \text{The}\;\text{states}\;\text{of}\;\text{channel}=\left\{close,\;open\right\} \end{array}$$

The left-hand-side of rules can trigger the right-side of rules when they satisfy the condition, which is fifth feature at the left-hand-side of rules. Each attribute has different condition value by its attribute type. For example:

$$\begin{array}{c} \text{The}\;\text{condition}\;\text{values}\;\text{of}\;\text{gene},\;\text{metabolite}=\left\{up,\;down\right\}\\ \text{The}\;\text{condition}\;\text{values}\;\text{of}\;\text{channel}=\left\{close,\;open\right\} \end{array}$$

The condition of a rule signifies the change of state value of an attribute.

The description of condition 'up' is:

*S_i_*(*t*) = state of *i *attribute gene at t step.

$${\text{S}}_{\text{i}}\left(t\right)=\left\{\begin{array}{cc} high, & S_{i}\left(t-1\right)=\left\{normal,\;high\right\}\\ normal, & {\text{S}}_{\text{i}}\left(t-1\right)=\left\{low\right\} \end{array}\right)$$

The description of condition 'down' is:

*S_i_*(*t*) = state of *i *attribute gene at t step.

$${\text{S}}_{\text{i}}\left(t\right)=\left\{\begin{array}{cc} normal, & S_{i}\left(t-1\right)=\left\{high\right\}\\ low, & {\text{S}}_{\text{i}}\left(t-1\right)=\left\{normal,\;low\right\} \end{array}\right)$$

When the condition is satisfied, the state of an attribute is changed by 'Action' in the right-hand-side of a rule. Each attribute has different action value by its attribute type. For example:

$$\begin{array}{c} \text{The}\;\text{condition}\;\text{values}\;\text{of}\;\text{gene},\;\text{metabolite}=\left\{up,\;down\right\}\\ \text{The}\;\text{condition}\;\text{values}\;\text{of}\;\text{channel}=\left\{close,\;open\right\} \end{array}$$

The description of action 'up' is:

*S_i_*(*t*) = state of *i *attribute gene at t step.

$${\text{S}}_{i}\left(t+1\right)=\left\{\begin{array}{cc} high, & S_{i}\left(t\right)=\left\{normal,high\right\}\\ normal, & {\text{S}}_{i}\left(t\right)=\left\{low\right\} \end{array}\right)$$

The description of action 'down' is:

*S_i_*(*t*) = state of *i *attribute gene at t step.

$${\text{S}}_{i}\left(t+1\right)=\left\{\begin{array}{cc} normal, & S_{i}\left(t\right)=\left\{high\right\}\\ low, & {\text{S}}_{i}\left(t\right)=\left\{normal,low\right\} \end{array}\right)$$

So, the description of this rule is that:

$$\left(\text{Cell},\;\text{hepatocyte},\;\text{gene},\;\text{PRKAB}1,\;\text{up}\right)\to \left(\text{Cell},\;\text{hepatocyte},\;\text{gene},\;\text{AGRP},\;\text{up}\right)$$

Gene AGRP, which is in hepatocyte cell, change state from 'normal', 'high' to 'high', or from 'low' to 'high' when gene PPKAB1, which is in hepatocyte cell, has changed state from 'normal', 'high' to 'high', or from 'low' to 'normal'.

### Rule extraction strategy

One of the main issues of system development is collecting rules of the system in a whole-body scale for the simulation. For this purpose, we have extracted rules from disparate information sources to increase coverage: pathway database, ordinary differential equation (ODE) model and Petri-net.

A rule curated from experts was described based on dynamic status rather than the static. It is based on the assumption that the body system changes its status through perturbation. If a certain component of cellular system does not alter, other components connected to it cannot be affected permanently. Thus, curators have extracted the rules from the context of the data that describes perturbed status of a certain component. A well-curated signalling pathway database, pathway interaction database (PID) provides valuable information of diverse signalling protein interactions. Regulations of proteins in each pathway were annotated explicitly, so it could be converted to our rule formulae straightforwardly. For the analysis of Type 2 diabetes (T2D), related pathways like insulin pathway and insulin-mediated glucose transport pathway in PID were considered to be extracted as a rule for the simulation. This database also provides integrated resources from external databases, such as BIOCARTA, REACTOME. Therefore, we could also collect other insulin pathway-related rules of external databases through these databases.

### Rule conversion from different modelling formalisms

An ODE model is composed of several differential equations describing relationship between variables in the model. Some of the variables have clear relationship (i.e., activation or inhibition) in, for instance, first-order linear differential equations. However, some of equations of nonlinear form make the relationship of variables difficult to determine.

We have examined whether the effect of a certain variable to others shows a monotonic changes. Taking an advantage of rule-based simulation, we have only focused on the direction rather than the quantity. We were able to extract rules of variables if they affect each other monotonically. We also identified the nonmonotonical relationship of variables if the range of changing pattern of effect could be determined.

Conventional Petri-net has the graphical structure containing a transition and a place as an element of the graph. A transition element means the changing action, not the actual component, that mediates the interconversion of place elements of the Petri-net through its action, and a place element is a component of the body system that takes the change of its status. Therefore, based on the context of transition elements, we were able to extract rules from places elements (i.e., place elements will be located in the left- and right-hand-side of a rule with its perturbed status determined by the context of transition elements.)

### The overview of simulation

**Figure 5:**
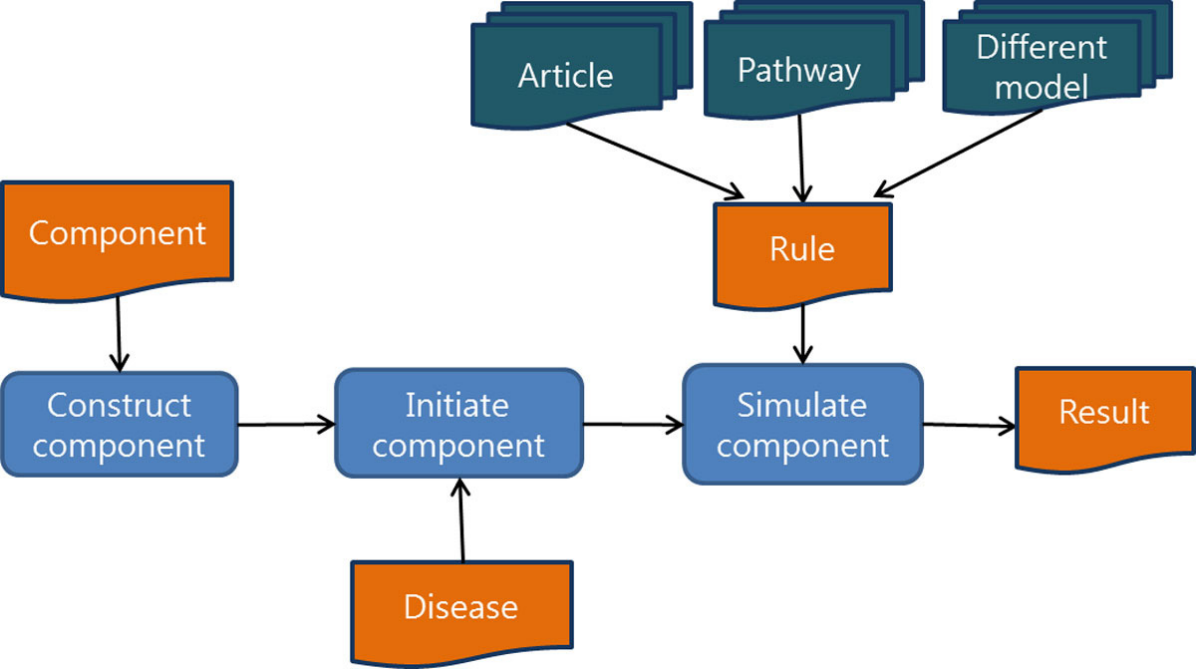
**The overview of system platform**. The platform constructs components by component file. Then, it initiates components by disease file, which contain components value in disease status. Then, it simulates component by rules.

This system has three kinds of inputs, such as a component, a rule, and a disease file. The component file contains information about components such as component types, component names, attribute types and attribute names. The system constructs components according to the component file (Figure 5). The disease file contains disease states of components. The system initiates the state of components by a disease file to make the system as a patient model. After all components in the system are initiated, the system simulate according to the rules in the rule file. The result file will be produced after simulation is over. The result file has all state changes of every component during the simulation. Each component has the threshold for changing its state. Each threshold of the components is different depending on the component types.

#### Simulation algorithm

This system uses an asynchronous updating simulation method to observe drug responses and drug effect pathways to implement stochastic rule firing. Before running simulation, the system is initialized by T2D patient model and T2D related drugs. Using T2D patient model, the states of components, which are related to T2D, in the system are changed to abnormal. Then, drugs are injected to the simulation system. The action of drug injection means the start of the simulation.

Algorithm.1 and 2 show the simulation running process. *R *means all rules in the system. *C *means all components in the system. *AR(t) *is a set that has all executable rules in the specific time t. *AC(t) *is a set which has all active components at time t. And *RFS(c) *is the rule firing score of a component c. After a rule execution, the *RFS *of a component, which is affected by the rule effect score, which is *RS(r)*, will be changed. *TH(c) *is the rule execution threshold of a component. If *RFS(c) *is greater than *TH*(c), the state of component c is updated by the rule.

To model differences in the speed of signal propagation in the body, we used a random-order asynchronous updating algorithm. Executable rules are selected through Algorithm 1. Next, one rule is randomly selected from executable rules and executed. Then, the states of components are updated if condition of the randomly selected rule is satisfied. Then, all rules activated by the randomly selected rule are added to *AR *for the next iteration step. We repeated the above simulation 100 times as a Monte Carlo approach for finding drug effect pathways.

*R *= {*x|x is a rule*}

*C *= {*x|x is a component*}

*AR*(*t*) = {*x|x is an active rule at time t*}

*AC*(*t*) = {*x|x is a component at time t*}

*RFS*(*c*) = (*rule firing score of a component*)

*TH*(*c*) = (*rule execution threshold of a component*)

*RS*(*r*) = (*rule effect score*)

*Algorithm*.1: ***RunSimulation***( )

*Algorithm*.2: ***UpdateComponentSet***(*AC*(*t*),*R_i_*)

Algorithm.2 shows how to update the states of the components in *AC *when $R_{i}$ is executed at time *t*. First, save all components affected by $R_{i}$ at $AC_{i}$. Then, repeat update of *RFS *of $C_{j}$ and check *RFS *is greater than threshold of the component $C_{j}$, which is *TH*, until *AC *has not any element. If the *RFS *is greater than the threshold, the state of the component is updated.

Algorithm.2 shows how to update the states of the components in *AC *when $R_{i}$ is executed at time *t*. First, save all components affected by $R_{i}$ at $AC_{i}$. Then, repeat update of *RFS *of $C_{j}$ and check *RFS *is greater than threshold of the component $C_{j}$, which is *TH*, until *AC *has not any element. If the *RFS *is greater than the threshold, the state of the component is updated.

#### Rule execution threshold

Real human body parts (i.e. organs, cellular components, enzymes) have biological functions which have various timescale to complete the function. Therefore, for more accurate simulation, each component has its own threshold that represents the state change. Each rule execution threshold of the components differs depending on the component type and attributes type.

We determined threshold of the components based on Bitting, et al [25] and assumed that molecules or cells have smaller threshold (1.0) than tissue or organ threshold (60.0).

## Competing interests

The authors declare that they have no competing interests.

## Authors' contributions

WH designed the method, validated results and wrote the manuscript, YH performed experiments and wrote the manuscript.SL extracted rules. DL supervised the study and revised the manuscripts. All authors reviewed and approved the manuscript.

## Declarations

The publication costs for this article were funded by the corresponding author.

This article has been published as part of *BMC Medical Informatics and Decision Making *Volume 13 Supplement 1, 2013: Proceedings of the ACM Sixth International Workshop on Data and Text Mining in Biomedical Informatics (DTMBio 2012). The full contents of the supplement are available online at http://www.biomedcentral.com/bmcmedinformdecismak/supplements/13/S1.
